# Spatial sampling bias and model complexity in stream‐based species distribution models: A case study of Paddlefish (*Polyodon spathula*) in the Arkansas River basin, USA

**DOI:** 10.1002/ece3.5913

**Published:** 2019-12-25

**Authors:** Andrew T. Taylor, Thomas Hafen, Colt T. Holley, Alin González, James M. Long

**Affiliations:** ^1^ Oklahoma Cooperative Fish and Wildlife Research Unit Department of Natural Resource Ecology and Management Oklahoma State University Stillwater OK USA; ^2^ U.S. Geological Survey Fort Peck Project Office Columbia Environmental Research Center Fort Peck MT USA; ^3^ U.S. Geological Survey Oklahoma Cooperative Fish and Wildlife Research Unit Department of Natural Resource Ecology and Management Oklahoma State University Stillwater OK USA

**Keywords:** conservation biology, ecological niche model, fisheries management, Maxent, riverscape ecology

## Abstract

Leveraging existing presence records and geospatial datasets, species distribution modeling has been widely applied to informing species conservation and restoration efforts. Maxent is one of the most popular modeling algorithms, yet recent research has demonstrated Maxent models are vulnerable to prediction errors related to spatial sampling bias and model complexity. Despite elevated rates of biodiversity imperilment in stream ecosystems, the application of Maxent models to stream networks has lagged, as has the availability of tools to address potential sources of error and calculate model evaluation metrics when modeling in nonraster environments (such as stream networks). Herein, we use Maxent and customized R code to estimate the potential distribution of paddlefish (*Polyodon spathula*) at a stream‐segment level within the Arkansas River basin, USA, while accounting for potential spatial sampling bias and model complexity. Filtering the presence data appeared to adequately remove an eastward, large‐river sampling bias that was evident within the unfiltered presence dataset. In particular, our novel riverscape filter provided a repeatable means of obtaining a relatively even coverage of presence data among watersheds and streams of varying sizes. The greatest differences in estimated distributions were observed among models constructed with default versus AIC_C_‐selected parameterization. Although all models had similarly high performance and evaluation metrics, the AIC_C_‐selected models were more inclusive of westward‐situated and smaller, headwater streams. Overall, our results solidified the importance of accounting for model complexity and spatial sampling bias in SDMs constructed within stream networks and provided a roadmap for future paddlefish restoration efforts in the study area.

## Graphical abstract

This study examines the effects of spatial sampling bias and model complexity on distribution estimates of the Paddlefish (*Polyodon spathula*) within a stream network. Available tools to address Maxent model complexity and performance currently rely on raster‐based input data; however, we provide customized R code (supplementary material, see Dryad) that allows exploration of these topics within stream segment‐based models. We hope this case study spurs development of tools that advance Maxent modeling practices within stream networks and other nonraster environments. Photograph by Brandon Brown, ODWC.

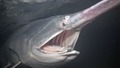

## 1 INTRODUCTION

Species distribution models (SDMs) are a powerful tool for informing biodiversity conservation. Using available species presence records and geospatial environmental data, researchers have constructed SDMs to estimate historic distributions, disentangle factors driving range loss, and explore how climate change might alter distributions (Elith et al., 2011; Guisan & Thuiller, 2005). Models built with biologically relevant predictor variables can identify the most influential variables in determining the distribution of species and estimate how habitat suitability for a species changes across a range of values (or categories) for a given variable (Elith et al., 2011). Resulting response curves and spatial distribution estimates can provide important baseline understanding of species ecology and overall conservation status. At present, Maxent is one of the most widely used distribution modeling algorithms among ecologists (Elith et al., 2011; Merow, Smith, & Silander, 2013; Phillips, Anderson, & Schapire, 2006). Maxent is a presence‐background algorithm that seeks to minimize the relative entropy between predictor variable values associated with known presence records and values associated with background samples from elsewhere within the study area by applying a number of predefined transformations to the predictor variables (Elith et al., 2011; Merow et al., 2013).

Maxent is generally considered one of the best performing presence‐only modeling algorithms (Elith et al., 2006; Pearson, Raxworthy, Nakamura, & Poret‐Peterson, 2007), yet concerns have emerged regarding potential sources of prediction error. For instance, Maxent users typically assume that sampling efforts and detection probabilities are equal across their study area; however, spatial sampling bias is commonplace when combining disparate presence data sources and can result in biased distribution estimates (Boria, Olson, Goodman, & Anderson, 2014; Kramer‐Schadt et al., 2013; Yackulic et al., 2013). Proposed methods to minimize the effects of spatial sampling bias include spatial filtering of presence records or manipulation of the background data to contain a similar spatial bias as the presence records (Dormann et al., 2007; Kramer‐Schadt et al., 2013; Merow et al., 2013). Other model‐based methods that have also been proposed to correct for spatial sampling bias methods exist, such as including known observer biases as covariates or incorporating information regarding sampling efforts or site accessibility (El‐Gabbas & Dormann, 2018; Warton, Renner, & Ramp, 2013). Another concern surrounds Maxent's default parameterization, which is prone to increased model complexity and overfitting that can lead to elevated omission error and poor transferability (Merow et al., 2013; Warren & Seifert, 2011). To account for model complexity, Maxent's regularization parameter can be sequentially increased, which reduces the number of model features and smooths fitted functions (Merow et al., 2013; Warren & Seifert, 2011). Warren and Seifert (2011) proposed that Akaike information criterion with small‐sample bias adjustment (AIC_C_; Akaike, 1973; Hurvich & Tsai, 1989) could be used to estimate the model of optimal complexity among a candidate set with varying levels of regularization. In recent years, a number of analytical packages in the R programming language (R Core Team, 2018) have been developed to streamline Maxent modeling workflows that account for spatial sampling bias (e.g., *spThin*; Aiello‐Lammens, Boria, Radosavljevic, Vilela, & Anderson, 2015) and model complexity (e.g., *ENMeval*; Muscarella et al., 2014), but these packages rarely consider the unique modeling environment that freshwater streams require.

The application of distribution modeling in freshwater stream systems to inform conservation action remains in its early stages relative to terrestrial systems (Liang, Fei, Ripy, Blandford, & Grossardt, 2013). The majority of studies using Maxent to model the distributions of stream species use raster data summarized at a coarse, watershed scale (for examples, see Cao et al., 2013; Liang et al., 2013) despite the increasing availability of finer‐resolution, stream segment‐based data in North America, like NHDplusV2 (Mckay et al., 2012) and StreamCat (Hill, Weber, Leibowitz, Olsen, & Thornbrugh, 2016). Relatively few studies have used these segment‐based geospatial datasets as the foundation for Maxent models for aquatic species (for example, see Dyer, Brewer, Worthington, & Bergey, 2013; Elith et al., 2011; Taylor, Papeş, & Long, 2018; Worthington, Brewer, Grabowski, & Mueller, 2014). We posit that one likely explanation for the lack of Maxent studies within stream networks is that segment‐based analyses require a tabular format (“samples‐with‐data” [SWD]) for data input rather than the conventional, visualization‐friendly approach of uploading multiple raster layers containing environmental covariate data. Unfortunately, many of the R packages containing functions to address model complexity and evaluate model performance are also built for raster‐based workflows (e.g., *ENMeval*; Muscarella et al., 2014), thus limiting the application of these concepts to models built within stream segments or other nonraster modeling environments.

Freshwater fishes and other aquatic organisms inhabiting streams face markedly high imperilment in North America and across the globe (Jelks et al., 2008; Olden et al., 2010), and distribution modeling could be beneficial to informing their conservation. For example, the paddlefish (*Polyodon spathula*) is a large‐bodied fish native to large rivers of the Mississippi River basin of North America (Jennings & Zigler, 2009) and is the subject of conservation efforts in many parts of its range. Habitat modification, fragmentation (i.e., dams), and overfishing (Bettoli, Kerns, & Scholten, 2009) have led to paddlefish range loss; however, paddlefish continue to support regulated commercial and recreational fisheries in portions of their former range. Because paddlefish migrate upstream for spawning, the closure dams could be preventing upstream spawning migrations to suitable habitats, including spawning grounds. In recent years, some paddlefish stocks have rebounded as a result of commercial fishing closures and restoration of extirpated populations (Bettoli et al., 2009).

Understanding the natural riverscape factors that influenced paddlefish distribution prior to the large‐scale habitat alteration could help prioritize future restoration efforts. For example, at a broader‐scale, paddlefish are commonly considered a “large‐river” fish, but the importance of stream size in influencing paddlefish habitat suitability, and how suitability varies across metrics related to stream size like mean annual discharge, both remain unknown. Paddlefish also require a certain set of finer‐scale environmental cues to complete their life cycle (Jennings & Zigler, 2009). In the spring, when water temperatures begin warming past 10°C, paddlefish begin to stage for spawning and ascend upstream from 20 km to over 100 km to spawn once a flood pulse begins (Firehammer & Scarnecchia, 2007; Lein & DeVries, 1998; Paukert & Fisher, 2001). Furthermore, paddlefish require a hard‐bottom substrate, such as gravel, for their eggs to adhere and develop (Jennings & Zigler, 2009; Purkett, 1961). Maxent models constructed at a broader, stream‐segment scale can identify the riverscape factors that influence paddlefish distribution and how each of those factors relate to paddlefish habitat suitability. Similarly, identifying suitable habitats at the segment scale can help direct site investigations of finer‐scale habitat conditions and assess the potential for successful reintroductions.

In this modeling exercise, we estimate the potential distribution of paddlefish (i.e., the abiotically suitable area) at the stream‐segment level within the Arkansas River basin, USA. In this area, habitat fragmentation by dams has led to suspected range loss of paddlefish, but there is active vested interest in restoring populations to potentially suitable environments. We account for potential spatial sampling bias within the available presence data by employing two spatial thinning methods, including a novel riverscape filter that accounts for watershed location and variation in stream size within watersheds. We also examine the effects of model complexity by comparing “full” Maxent models (default regularization) to AIC_C_‐selected models with increased regularization, complete with common model evaluation metrics. We provide an R script of the workflow for these modeling steps with a nonraster dataset, which may be useful to other researchers interested in the effects of model complexity on Maxent predictions within stream systems. Results of this study can be used to better understand the environmental factors influencing paddlefish habitat suitability and identify stream reaches for potential restoration.

## 2 METHODS

### 2.1 Study area

**Figure 1:**
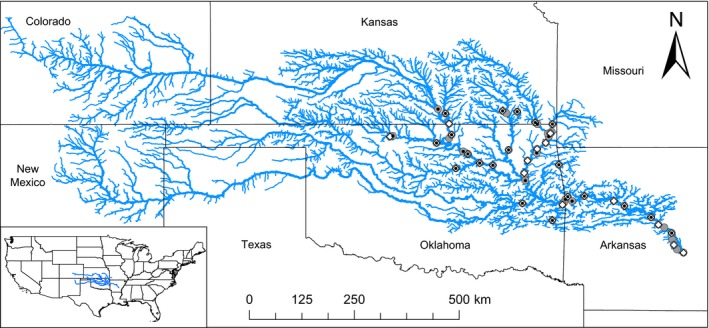
The Arkansas River basin shown with the subsets of paddlefish presence data used in modeling. Blue lines represent National Hydrography Dataset (NHD) flowlines wherein line widths increase with stream order. Gray circles represent presence locations from the unfiltered dataset, white diamonds represent the distance filter, and black dots represent the riverscape filter

The Arkansas River basin (Figure 1) encompasses 409,273 km^2^ across seven states (Colorado, New Mexico, Texas, Kansas, Oklahoma, Missouri, and Arkansas) with diverse geography and a west‐to‐east precipitation and temperature gradient. The cooler headwaters begin at the continental divide, at 4,300 m elevation with snowfall‐driven precipitation averaging 1,020 mm annually, driving the hydrology for the western region (Cain, 1987). As the headwaters converge, the elevation decreases to 1,020 m, the topography gradually changes from mountains to plains and precipitation drops to 250 mm annual average (Cain, 1987). Moving eastward across the basin, igneous and metamorphic mountains transition to plains and the geology changes to bedrock and sedimentary rock and the river becomes a plains river (Cain, 1987). Hydrology in the plains is driven more by rainfall from summer thunderstorms than snowfall. In the eastern portion of the basin, water is diverted and dammed for irrigation and navigation; for instance; the mainstem of the Arkansas River alone has 13 locks and dams. Streamflow along the mainstem is regulated until it reaches the confluence with the Mississippi River (Burns, 1985).

The NHDplusV2 dataset (Mckay et al., 2012) is a vector‐based representation of river networks and their associated watershed boundaries, with individual stream segments delineated at each junction with another stream. Within the NHDplusV2 dataset (Mckay et al., 2012), we defined our study extent as the Arkansas River basin within the HUC 2‐digit basin 11, excluding the adjacent Red and White river drainages. The spatial grain was defined as individual stream segments that were uniquely identified via the COMID attribute.

### 2.2 Input data

#### 2.2.1 Presence records

Eighty‐nine presence records were compiled from several sources including GBIF (http://www.gbif.org), MARIS (http://www.marisdata.org), and several publications (Bostian, 2015; Leone, Stoeckel, & Quinn, 2012; Long, Schooley, & Paukert, 2017; Neely, Steffen, Lynott, & Koch, 2015a, 2015b; Paukert & Fisher, 2000, 2001; Riggs & Moore, 1949; Robison & Buchanan, 1988; Schooley & Johnston, 2015). We cleaned these records by removing duplicate records that contained the exact same coordinates or locality descriptions, and we removed records that featured vague locality descriptions that precluded accurate geospatial referencing to a stream system. Records that lacked coordinates but featured descriptive locality information were georeferenced with GEOLocate v. 3.22 (Rios & Bart, 2010) to the nearest stream. Presence records were imported into ArcMap v.10.4 (ESRI) wherein coordinates were linked to the nearest stream segment using a spatial join. We then compared the linked NHDplusV2 attributes (e.g., stream name) to the presence record data (e.g., locality description) to ensure the joining procedure was accurate. The resulting full dataset contained 51 unique records spanning from 1927 to 2018 that represented 49 unique stream segments (Figure 1).

#### 2.2.2 Environmental covariates

Distribution model covariates within the Arkansas River basin were selected based on biological relevance to paddlefish and obtained from NHDplusV2 (Mckay et al., 2012) and StreamCat (Hill et al., 2016) databases, both of which have linked a number of environmental covariates (such as geology, hydrology, and elevation) to each NHD plusV2 segment or its contributing watershed(s). We considered a number of abiotic covariates that characterized natural stream gradients, network connectivity, and geology (Table 1). Natural gradients in stream size, discharge, temperature, elevation, and slope are fundamental in determining the distribution of aquatic fauna within riverscapes (Vannote, Minshall, Cummins, Sedell, & Cushing, 1980). Connectivity also influences the distribution of fishes within stream networks, and differences in confluence size can be particularly important for migratory species like paddlefish (Fullerton et al., 2010). Underlying geology influences the physicochemical properties of streams (Hynes, 1975); for example, watersheds containing a calcareous geology have a buffering capacity that generally supports increased biomass of aquatic organisms (Pyne, Rader, & Christensen, 2007). Geology can also influence the distribution of suitable spawning substrates, like exposed bedrock or gravel, within stream networks. Covariates were incorporated at one of three relevant spatial scales within the hierarchical structure of stream networks: stream segment, local catchment, or full watershed (Domisch, Jähnig, Simaika, Kuemmerlen, & Stoll, 2015). Each covariate was associated with the unique COMID identifier for individual stream segments in the study area. To avoid issues arising from multicollinearity among covariates, we calculated Pearson's correlation coefficients (*r*) and manually selected a subset of relevant covariates wherein |*r*| ≤ .70 for model construction based on ecological relevance and hypothesized mechanisms (Dormann et al., 2013).

**Table 1:** Environmental covariates used to model the potential distribution of paddlefish in the Arkansas River basin, USA, and whether or not the covariate was used in the final models after removing high intercorrelations

Abbreviation	Covariate name	Mean	Min.	Max.	Unit	Scale	Source	Included?
SLOPE	Slope	0	0	1	–	Segment	NHDplusV2	Yes
MAXELEVSMO	Maximum elevation	5,540	63,271	396,264	cm	Segment	NHDplusV2	Yes
SandCat	Mean % sand content of soils	26	5	83	%	Catchment	StreamCat	Yes
RckDepCat	Mean depth of bedrock of soils	127	42	152	cm	Catchment	StreamCat	Yes
Q0001C_Yr	Mean annual discharge	4	0	1,279	m^3^/s	Segment	NHDplusV2	Yes
DSMainLinkSize	Downstream mainstem link stream order	4	1	9	–	Segment	NHDplusV2	Yes
CaOCat	Mean % lithological calcium oxide in surface	10	0	48	%	Catchment	StreamCat	Yes
StreamOrder	Stream order	2	1	9	–	Segment	NHDplusV2	
TotDASqKM	Total Drainage Area	3,393	0	397,422	km^2^	Segment	NHDplusV2	
Precip8110Ws	30‐year normal mean precipitation	857	248	1,734	mm	Watershed	StreamCat	
Tmin8110Ws	30‐year normal mean minimum temperature	7	−10	12	°C	Watershed	StreamCat	
Tmean8110Ws	30‐year normal mean temperature	14	−3	18	°C	Watershed	StreamCat	
Tmax8110Ws	30‐year normal maximum temperature	20	3	23	°C	Watershed	StreamCat	

### 2.3 Distribution modeling

#### 2.3.1 Basic settings

We used a presence‐background approach in Maxent (Phillips & Dudík, 2008) to estimate the historic distribution of paddlefish within the Arkansas River basin and to examine the relationships between paddlefish presence and environmental covariates. As a machine‐learning tool, Maxent minimizes the relative entropy between values of environmental covariates associated with known presence locations and values of environmental covariates associated with background samples within the study area (Elith et al., 2011; Phillips et al., 2006). Maxent models were constructed with the R programming language (v.3.5.1; R Core Team, 2018) using the *dismo* package (v. 1.1‐4; Hijmans, Phillips, Leathwick, & Elith, 2017) and the “maxent” command to call the maxent.jar executable file (v. 3.4.1; Phillips, Anderson, Dudík, Schapire, & Blair, 2017; Phillips & Dudík, 2008). Because our models are constructed without raster input, we used the SWD format to create our input files. We adopted the cloglog transformation of Maxent's raw output as a readily interpretable index of habitat suitability ranging from zero to one (Phillips et al., 2017). Arguments were specified to alter Maxent settings (Phillips et al., 2006); for example, we enabled the “removed duplicates” function as an additional data quality filter (i.e., preventing any segment from being represented more than once in modeling), we allowed partial environmental covariate coverage of presence locations, and we set the number of background locations so that all stream segments (*n* = 126,422) were included instead of a random sample. Additionally, we created a placeholder so that the beta multiplier (*β*) argument could be easily manipulated across model runs (see Section 2.3.3 for more detail). For each model, we used the percent contribution (path‐dependent) and the permutation importance (final model importance) as output by Maxent to assess the relative importance of each covariate to model gain. We saved the final Maxent prediction for each stream segment using the “project” command within *rmaxent* (v.0.8.3.9000; Baumgartner, Wilson, & Esperon‐Rodriguez 2017).

#### 2.3.2 Spatial sampling bias

Upon plotting the complete (i.e., unfiltered) Paddlefish presence dataset, the majority of presence records appeared to be congregated along the farthest downstream reaches of the study area (Figure 1). In this case, the uneven distribution of records likely represented spatial sampling bias related to sampling access (Boria et al., 2014). To reduce the potential effects of spatial sampling bias on model results, we applied a distance filter and a novel riverscape filter to our full presence dataset. Filtering can dampen the influences of spatial sampling bias, although potential drawbacks are that the size of the filter is commonly subjective and the presence records that are removed likely reflect suitable environments (Feng, Anacleto, & Papeş, 2017; Fourcade, Engler, Rödder, & Secondi, 2014). A distance filter was performed in the *spThin* package in R (Aiello‐Lammens et al., 2015), which retained the maximum number of records ≥20 km apart in straight‐line, aerial distance. This resulted in 32 records, each representing a unique stream segment. For the riverscape filter, which is philosophically similar to an environmental filter (Varela, Anderson, García‐Valdés, & Fernández‐González, 2014), we sought to more evenly represent the spatial distribution of records (among Hydrologic Unit Code 8‐digit [HUC8] watersheds) and the distribution of records across stream sizes (stream order) by retaining one record from each unique HUC8‐by‐stream order combination. For example, the unfiltered dataset (51 presence records) had seven records in HUC 11110207, but all within a ninth‐order stream so we haphazardly chose one of these records to retain in the trimmed dataset. In contrast, HUC 11060006 contained three records and we retained all three because they each represented different stream orders (third, sixth, and eighth). The remaining dataset resulted in 29 records, each representing a unique stream segment. We constructed independent models with each of the three presence datasets (unfiltered, distance filter, and riverscape filter).

#### 2.3.3 Model complexity

For each presence dataset, we constructed models with varying levels of complexity to explore how model overfitting could influence estimated distributions. Specifically, we adjusted the *β* multiplier (also known as the regularization multiplier), a parameter that acts across all feature classes (as defined by the “autofeature” setting) as a coefficient that is multiplied to the specific regularization values (i.e., the *β*'s) associated with each feature class. We allowed the *β* multiplier to vary between 1.0 (default parameterization) and 5.0 by intervals of 0.5 (sensu Merow et al., 2013; Guevara, Gerstner, Kass, & Anderson, 2018). In all models, the “autofeature” option was enabled wherein Maxent automatically limits which feature classes (of linear, quadratic, product, and hinge feature options) were used based on the size and threshold of the training dataset. Therefore, as the *β* multiplier is increased, Maxent's settings begin to constrain overparameterization, both in the number of feature classes included in the model and in the smoothness of fitted features (Elith et al., 2011; Merow et al., 2013; Warren & Seifert, 2011). To compare models of differing complexity, we report results from the default parameterization and the model of optimal complexity as approximated by AIC_C_ selection (Warren & Seifert, 2011). Briefly, AIC_C_ was calculated by estimating the number of nonzero parameters within each model's.lambda file and based on the predicted values across the entire sample of background stream segments (sensu Warren & Seifert, 2011).

#### 2.3.4 Model evaluation

Model evaluation metrics were calculated similar to the *ENMeval* package for R (Muscarella et al., 2014); however, this package relies on raster‐formatted input data, necessitating us to write R code to calculate model evaluation metrics. For each model considered, we conducted a fivefold cross‐validation wherein presence records were randomly partitioned into testing and training sets, and metrics of model performance were then calculated as the average across folds. The receiver operating characteristic area under the curve (ROC AUC) is a threshold‐independent measure of model performance (Fielding & Bell, 1997), so we calculated AUC_TEST_ as in Muscarella et al. (2014). Higher values of AUC_TEST_ reflect an improved ability to discriminate at testing locations compared with background locations (Muscarella et al., 2014; Warren & Seifert, 2011). In addition, we adopted a threshold‐dependent measure to further assess the discrimination capacity of models (Jiménez‐Valverde, 2014) by calculating OR_MTP_, the average omission rate of the testing records at the minimum training presence (MTP) threshold (i.e., the lowest Maxent predicted value associated with a training record). The MTP threshold represents an inclusive estimate of species habitat suitability (Anderson & Gonzalez, 2011).

### 2.4 Comparing models

We examined a total of six final models to evaluate the potential effects of spatial sampling bias (unfiltered, distance filter, and riverscape filter) and model complexity (default and AIC_C_‐selected parameterizations). Paddlefish distribution estimates were plotted in ArcMap using the MTP threshold to map the segments predicted suitable by each model. Differences in the number of stream segments considered suitable were calculated across spatial bias and model complexity groupings. Model differences were also quantified by two measures of niche similarity, Schoener's *D* and Warren's *I* (Schoener, 1968; Warren, Glor, & Turelli, 2008), that were calculated in a pairwise fashion based on segment‐level model estimates. The percent contribution and permutation importance of each environmental covariate was compared across models to assess any changes in the relative importance of predictor variables. We plotted single‐variable response curves (Phillips, 2005) to examine species‐habitat relationships for covariates with >50% contribution averaged across all six models. Model evaluation metrics were compared with determine whether discrimination capacity varied markedly across models. Finally, we created an ensemble distribution estimate by calculating the sum of models (from 0 to 6) that estimated paddlefish presence at the MTP across unique stream segments, thus visualizing how consistently each segment was estimated as suitable. An ensemble approach recognizes that each model may be flawed, but all provide useful information (Araújo & New, 2007). In our case, an ensemble distribution estimate can help identify stream segments that are consistently predicted suitable and thus represent the best targets for paddlefish restoration.

## 3 RESULTS

**Figure 2:**
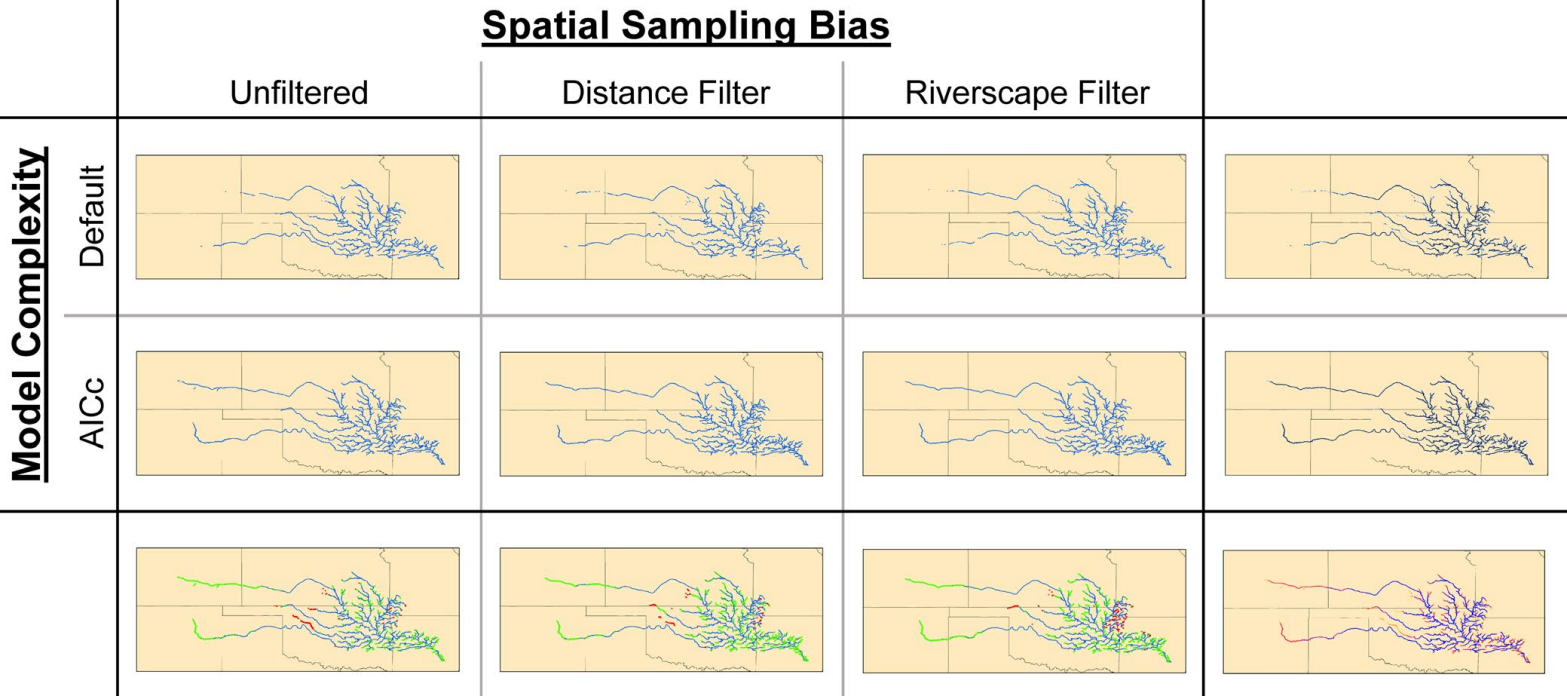
Paddlefish potential distribution in the Arkansas River basin, USA, based on the minimum training presence (MTP) threshold, as estimated across three presence datasets (to account for spatial sampling bias) and two model complexities. Bottom row features comparisons between the default and AIC_C_‐selected models for a given presence dataset, wherein green segments were gained in the AICc model and red segments were lost. Right‐hand column illustrates agreement across the three presence datasets for a given model complexity, wherein darker shades indicate the highest agreement. The bottom, right‐hand cell is an ensemble map illustrating areas that were consistently estimated suitable among the six models

**Figure 3:**
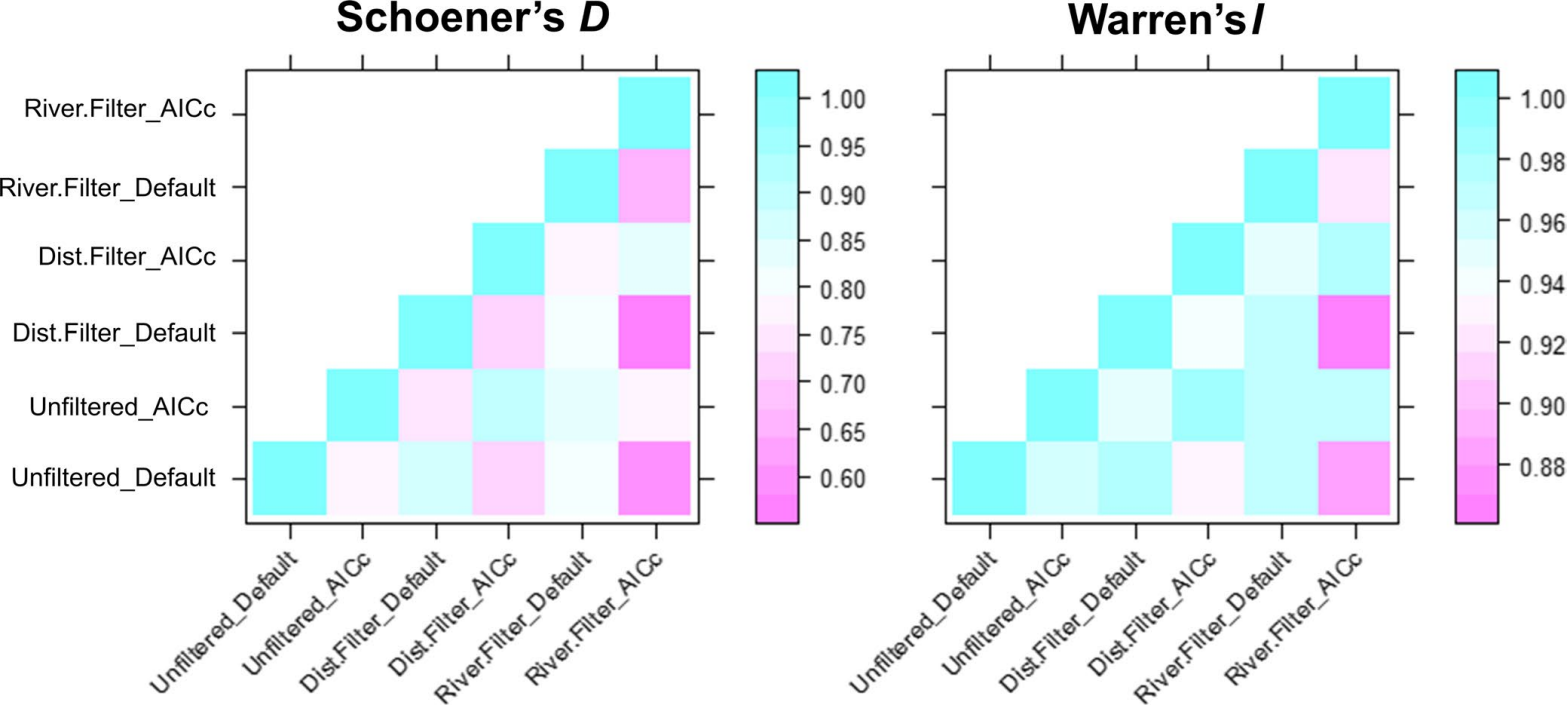
Heatmaps illustrating two pairwise comparisons of niche similarity, Schoener's *D* and Warren's *I*, for models of paddlefish distribution in the Arkansas River basin, USA.

**Figure 4:**
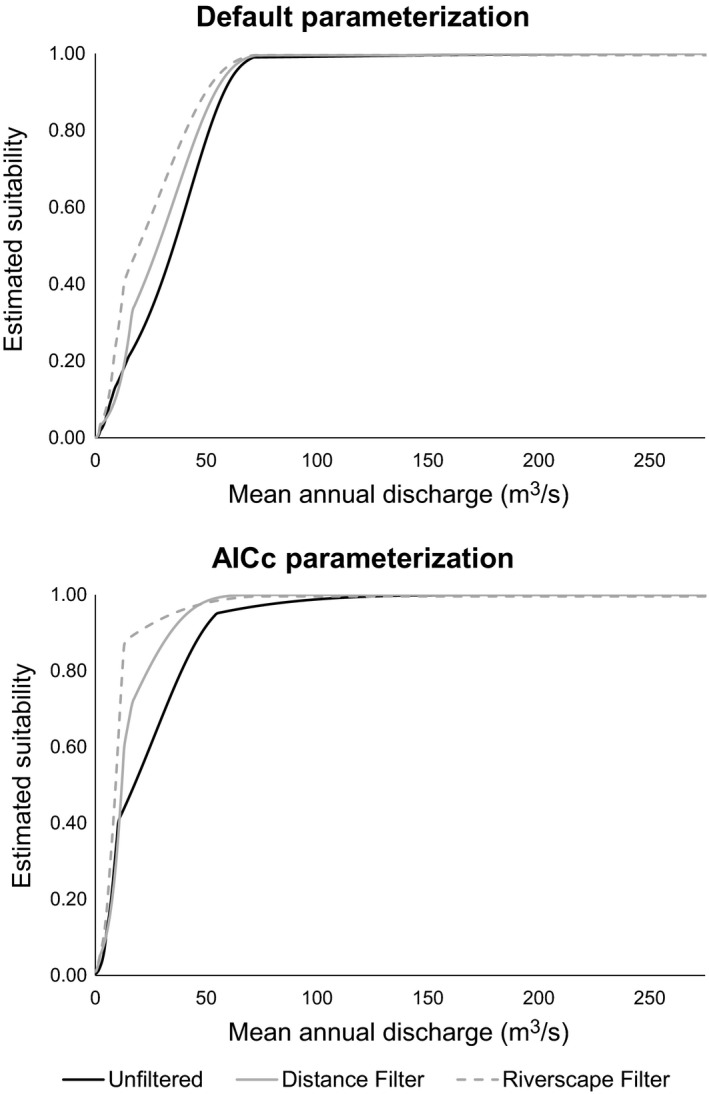
Single‐variable response curves relating estimated paddlefish habitat suitability (i.e., Maxent's cloglog output) to mean annual discharge (m^3^/s) in the Arkansas River basin, USA. Unfiltered presence locations were associated with a mean of 357 m^3^/s, a median of 87 m^3^/s, and a range of 2–1,279 m^3^/s in mean annual discharge

The six paddlefish distribution models we examined shared some overarching commonalities. In general, all models estimated elevated paddlefish habitat suitability in larger, more easterly streams in the Arkansas River basin (Figure 2). Pairwise calculations of Schoener's *D* varied from 0.593 to 0.896 and Warren's *I* varied from 0.873 to 0.991, signifying high levels of similarity (Figure 3). Mean annual discharge contributed the most to model gain in all six models (overall mean of 96.0% percent contribution and 96.3% permutation importance; Table 2), and the response curves relating suitability to mean annual discharge generally depicted a logistic response wherein suitability was initially low at lower mean annual discharge, but suitability approached 1.000 as mean annual discharge surpassed 56 m^3^/s (Figure 4). Maximum elevation and downstream link size contributed much less to model gain (means of 1.4% and 1.2%, percent contribution and means of 2.8% and 0.3% permutation importance, respectively), wherein suitability was negatively related to maximum elevation and positively related to downstream link size. Model evaluation metrics indicated that all models performed well, as AUC_TEST_ ranged from 0.986 to 0.993 (wherein a value of 1.000 would indicate perfect discriminative ability) and OR_MTP_ ranged from 0.020 to 0.044 (slightly elevated from the expected omission rate of 0.000; Table 3).

**Table 2:** Percent contribution and permutation importance of environmental covariates to gain of models of paddlefish distribution in the Arkansas River basin, USA, as organized from highest‐to‐lowest contributing by the overall average contribution across six models

	Full		Spatial		Riverscape		Overall
	Default	AICc	Default	AICc	Default	AICc	Average
Percent contribution							
Q0001C_Yr	95.9	95.8	96.2	97.1	93.5	97.5	96.0
MAXELEVSMO	0.6	0.8	0.8	2.0	1.5	2.5	1.4
DSMainLinkSize	0.7	2.9	0.0	0.9	2.8	0.1	1.2
RckDepCat	1.5	0.0	1.9	0.0	0.2	0.0	0.6
CaOCat	0.7	0.2	1.0	0.0	0.7	0.0	0.4
SandCat	0.4	0.2	0.2	0.0	0.5	0.0	0.2
SLOPE	0.2	0.0	0.0	0.0	0.8	0.0	0.2
Permutation importance							
Q0001C_Yr	96.0	97.9	95.7	96.3	94.9	97.0	96.3
MAXELEVSMO	2.6	1.3	3.4	2.9	4.3	2.4	2.8
DSMainLinkSize	0.3	0.0	0.0	0.6	0.1	0.6	0.3
SandCat	0.5	0.2	0.5	0	0.3	0.0	0.3
RckDepCat	0.3	0.2	0.3	0	0.1	0.0	0.2
CaOCat	0.1	0.4	0.1	0.1	0.0	0.0	0.1
SLOPE	0.3	0.0	0.0	0	0.3	0.0	0.1

**Table 3:** Evaluation metrics of models of paddlefish distribution in the Arkansas River basin, USA, as compared across three presence datasets (unfiltered, distance filter, and riverscape filter) and across varying levels of model complexity (default vs. AIC_C_‐selected parameterizations)

Presence dataset	Model	*β*	AUC_TEST_	OR_MTP_	Params	LogL	AIC_C_
Unfiltered	AIC_C_	4.5	0.991	0.020	13	−395.389	827.178
(*n* = 49)	Default	1.0	0.993	0.044	30	−380.506	924.345
Distance filter	AIC_C_	4.5	0.990	0.029	9	−261.926	550.033
(*n* = 32)	Default	1.0	0.992	0.033	24	−264.499	748.426
Riverscape filter	AIC_C_	5.0	0.986	0.040	9	−258.312	544.097
(*n* = 29)	Default	1.0	0.990	0.033	29	−266.888	a

a Models with more parameters than data points violate assumptions of AIC_C_ (Warren & Seifert, 2011).

Despite these commonalities, differences in the distribution models were evident when comparing models built with the three different presence datasets to address spatial sampling bias. The distance and riverscape filtering methods estimated suitability farther west (e.g., Colorado and New Mexico) than did the unfiltered dataset, suggesting that both filtering methods dampened the effects of the potential spatial sampling bias in the unfiltered dataset (Figure 2). The distance and riverscape filtering methods also predicted suitability that dispersed into smaller streams than models built with the unfiltered dataset. With the default parameterization, 4% of all segments in the study area were estimated suitable by models built with each of the three presence datasets, whereas 6% of segments were estimated suitable by all three models with the AIC_C_‐selected parameterizations. Regardless of model complexity, niche similarity metrics demonstrated that models built with the unfiltered presence dataset differed most with models built with the riverscape filtered dataset, with the spatially thinned dataset as an intermediate. These differences were also evident among the response curves, wherein models built with the riverscape filtered dataset estimated higher suitability at lower mean annual discharge values than did models built with the unfiltered dataset (Figure 4). Across both levels of model complexity, AUC_TEST_ was consistently highest for the full dataset, followed by the spatially thinned dataset and the riverscape filtered dataset (which also corresponded with decreasing number of presence records; Table 3). However, a similar trend was not evident for OR_MTP_ values, suggesting that all models had similar discrimination capabilities.

Differences in model complexity, as compared between using the default parameterization (*β* = 1.0) versus AIC_C_‐selected parameterization (*β* = 4.5–5.0, the maximum we explored in our study; Table 3), resulted in models that differed in subtle, yet important ways. There was never more than a 3% difference in the number of segments estimated suitable between the two parameterizations (for any of the three presence datasets); however, these differences resulted in noticeably spatial distributions (Figure 2). For example, distributions estimated with the default parameterization featured spatially disjunct segments, which could indicate model overfitting, whereas distributions estimated with an AIC_C_‐selected parameterization had more contiguity among segments estimated as suitable. Models with AIC_C_‐selected parameterization also estimated suitability farther upstream than did default parameterizations, particularly in western regions. As such, the greatest differences in niche similarity metrics were found when comparing models with AIC_C_‐selected parameterization (i.e., more inclusive distribution estimates) to models built with the default parameterization (i.e., more restricted distribution estimates; Figure 2). Models with AIC_C_‐selected parameterization contained 13 parameters (features) at most, compared with 30 at most among the default parameterizations, which resulted in more generalized or inclusive models. A smoothing effect of elevated *β* is demonstrated when comparing the response curves relating suitability to mean annual discharge (Figure 4). With default parameterization, suitability increased to a plateau at approximately 70.8 m^3^/s (Figure 4 top) whereas the plateau with AIC_C_‐selected parameters (i.e., elevated *β*) peaked quicker at approximately 42.5–50.9 m^3^/s (Figure 4 bottom), resulting in more, smaller stream segments estimated as suitable for paddlefish. In terms of model evaluation metrics, AUC_TEST_ was consistently higher for default parameterizations, yet OR_MTP_ was also higher for default parameterizations in two of the three presence datasets, indicating the default models may be overfit as compared with the AIC_C_‐selected models (Table 3).

The ensemble distribution map (Figure 2) visualized how consistently each stream segment was estimated as suitable at the MTP across the six models. Several large river systems, including large sections of the Arkansas, Canadian, and Cimarron rivers, featured a west‐to‐east gradient of increased agreement among the six models. In general, larger streams were more consistently considered suitable for paddlefish compared with upper reaches of smaller streams. Contiguous sections of stream that were consistently estimated as suitable across all six models, but currently lack paddlefish, represent the most promising areas for future targeted restoration based on our modeling efforts.

## 4 DISCUSSION

This study explored the influences of spatial sampling bias and model complexity on SDMs for paddlefish in the Arkansas River basin, which, to the authors’ collective knowledge, is one of the first studies to explore the effects of these widely recognized sources of bias in Maxent models constructed within a stream segment network. Filtering the presence dataset appeared to address initial concerns about an eastward, large‐river sampling bias within the full presence dataset. In particular, the novel riverscape filter may be useful for future modeling efforts in streams because it provides a repeatable means to ensure spatial coverage of presence data among watersheds and streams of varying sizes. The greatest differences in estimated distributions, however, were observed between models constructed with default versus AIC_C_‐selected parameterization. Although all models had similarly high performance and evaluation metrics, the AIC_C_‐selected models were more inclusive of westward‐situated and smaller, headwater streams. Overall, our results solidified the importance of accounting for model complexity and spatial sampling bias in SDMs constructed within stream networks while also informing future paddlefish restoration efforts in our study area.

Spatial sampling bias is a widely recognized issue within the SDM literature wherein areas oversampled in geographic space may result in models overfit to those biases in environmental covariate space (Boria et al., 2014). In stream networks, accounting for spatial sampling bias may be particularly pertinent because differences in sampling accessibility and methodologies are often related to environmental factors like stream size, depth, elevation, and proximity to public access points like bridges or boat ramps (Murphy & Willis, 1996). Furthermore, many stream fishes have migratory life histories that could result in a biased representation of their overall distribution within a stream network. For example, paddlefish may only use smaller streams during infrequent windows of high discharge during the spawning season each year (Jennings & Zigler, 2009; Lein & DeVries, 1998), perhaps making them less likely to be documented in those areas compared with larger streams where they may occur more regularly. To minimize the effects of spatial sampling bias, researchers often perform distance‐based filtering of presence records or manipulate the background data to contain a similar spatial bias as the presence records (Dormann et al., 2007; Kramer‐Schadt et al., 2013; Merow et al., 2013). Distance filtering appears to be the more commonly applied technique because it does not require the creation of a bias file based on relative sampling effort or density of presence records (Kramer‐Schadt et al., 2013). Unfortunately, filtering methods necessitate the loss of presence data from the training set, resulting in models that may be informative (even with as little as 15 records; Støa, Halvorsen, Stokland, & Gusarov, 2019), but with more weight placed on each of the remaining records. As such, care is needed to filter presence data in meaningful ways. Distance filtering is common practice in terrestrial settings (Boria et al., 2014), but this method often lacks a biological justification for the aerial distance used (e.g., home range size) and does not consider riverscape network position (e.g., two records situated in neighboring headwater streams may be situated within a 20‐km aerial distance, but may be separated by a large watershed boundary). For these reasons, we suggest that our novel riverscape filter could be useful in minimizing spatial sampling bias concerns within stream networks, particularly when sampling effort or accessibility varies with stream size.

At first glance, the estimated distributions produced may seem overly broad in comparison with presence locations used to build each model, especially for models built with AIC_C_‐selected parameterizations. Research with virtual species has shown that AIC_C_‐selected models tend to overpredict, with larger commission and omission errors compared with models that do not use AIC_C_ (Velasco & González‐Salazar, 2019). But, for our purposes of discovering potentially suitable areas for paddlefish restoration, producing a map that might overpredict habitat suitability is not necessarily bad. The migratory nature of our study species also likely influenced the estimated distributions by including some records that are representative of spawning migrations into smaller streams. Paddlefish may ascend over 100 km upstream to spawn in the spring when river discharges increase (Firehammer & Scarnecchia, 2007; Lein & DeVries, 1998; Paukert & Fisher, 2001), with some smaller rivers becoming suitable for spawning in specific years as a result of variation in rainfall‐induced flood pulses (Jennings & Zigler, 2009). Because paddlefish migrations likely correspond to environmental conditions fluctuating at finer spatial and temporal scales than could be incorporated into our modeling efforts, some weakening of species‐environment relationships is expected (McPherson & Jetz, 2007). Thus, the dynamic migratory nature of paddlefish likely resulted in mild overprediction of habitat suitability in westward‐positioned and headwater stream reaches.

This modeling exercise provided some of the first quantitative estimates of paddlefish habitat suitability, and the ensemble model identified promising sections of stream for future restoration efforts. Although paddlefish have long been regarded as a “large‐river” fish (Jennings & Zigler, 2009), results from our modeling exercise confirmed the importance of discharge and visualized the range in mean annual discharge that confers highest habitat suitability in our study area. Through our study, we addressed two major sources of potential model bias that can inflate omission error, commission error, or both: spatial sampling bias (Boria et al., 2014; Kramer‐Schadt et al., 2013; Yackulic et al., 2013) and model complexity (Merow et al., 2013; Velasco & González‐Salazar, 2019; Warren & Seifert, 2011). Although these sources of model error are often recognized, modelers typically lack the independent testing data needed to fine‐tune a predictive model to optimal settings (e.g., Fielding & Bell, 1997). In cases without independent testing data, such as our own, an ensemble model created across varying conditions can identify stream segments that were consistently estimated as suitable. Recent paddlefish restoration efforts in Oklahoma have focused on stocking impoundments within larger river systems, but these efforts have been met with disparate results. For example, Oologah Lake on the Verdigris River was stocked from 1995 to 2000 and has since shown signs of natural recruitment, whereas Lake Texoma on the Red River (outside our study area) was stocked from 1997 to 2007 but has not evidenced natural recruitment (Patterson, 2009, J. Schooley, ODWC, personal communication). The exact mechanisms behind this variation in restoration success remain unknown, but the hydrology and availability of suitable spawning habitat in upstream tributaries is considered key (Patterson, 2009; Paukert & Fisher, 1998; Schooley & Neely, 2018). Our ensemble map provided a visualization of stream reaches that were estimated as suitable for paddlefish. Focusing restoration efforts on stream reaches between dams and other barriers that contain interconnected segments that were consistently estimated as suitable could increase the likelihood of successful restoration.

Conservation of stream fishes has long been hindered by a limited understanding of species‐habitat relationships and species responses to anthropogenic alterations within stream networks (Jelks et al., 2008). With existing presence records and a wealth of geospatial data already linked to stream segments (e.g., Hill et al., 2016; Mckay et al., 2012), species distribution models represent an accessible and informative first step in advancing conservation and restoration of stream fishes (Taylor et al., 2018; Worthington et al., 2014). Although the application and advancement of Maxent models within stream networks has lagged behind those built in raster‐based (e.g., terrestrial) environments, we hope this case study inspires future advancements in species distribution modeling within stream networks. In particular, there is a need to develop model evaluation tools, like *ENMeval*, that accept standard data frames as data input towards providing repeatable methods to account for potential sources for prediction errors in stream networks and other nonraster environments.

## CONFLICT OF INTEREST

The authors declare they have no conflict of interest.

## AUTHOR CONTRIBUTIONS

A.T. led a modeling course that kick‐started the idea for this project. All authors contributed to the design and implementation of the research, analysis of results, and writing the manuscript. A.T. and C.H. created tables and figures.

## PERMITS

No permits were required during the course of this study.

## Data Availability

The presence data, environmental data, R code, and Maxent outputs that were generated during this study are available on Dryad at https://doi.org/10.5061/dryad.d7wm37px9.
